# An In-Depth Look Into the Epidemiological and Etiological Aspects of Prostate Cancer: A Literature Review

**DOI:** 10.7759/cureus.48252

**Published:** 2023-11-04

**Authors:** Munir Al-Ghazawi, Hamza Salameh, Samuel Amo-Afful, Seren Khasawneh, Rami Ghanem

**Affiliations:** 1 Urology, Barts Health National Health Service (NHS) Trust, London, GBR; 2 Orthopedics, North Devon District Hospital, Barnstaple, GBR; 3 Urology, Dorset County Hospital, Dorset, GBR; 4 Dentistry, Jordan University Hospital, Amman, JOR; 5 Urology, King Hussein Cancer Center, Amman, JOR

**Keywords:** prostate cancer screening, public health, cancer prevention, urology, prostate cancer

## Abstract

Prostate cancer is the second most frequently diagnosed cancer among men worldwide, and it represents a substantial worldwide health issue, primarily impacting men as they grow older. Understanding its epidemiology and etiology is crucial for crafting efficient preventive measures and enhancing treatment results. The epidemiology of this disease provides valuable insights into its prevalence and distribution. Age is a critical factor, with the risk of prostate cancer increasing with advancing years. Incidence rates are notably higher in developed countries, suggesting a role for lifestyle and environmental factors. Furthermore, there are significant racial and geographical disparities in prostate cancer incidence, with African-American men experiencing both a higher incidence and more aggressive forms of the disease. On the other hand, hormones, especially testosterone and its conversion to dihydrotestosterone (DHT), contribute to prostate cell growth and, potentially, cancer. Genetics also plays a pivotal role, with certain gene mutations, like Breast Cancer gene 1 & 2 (BRCA1 and BRCA2), elevating risk. Dietary habits and lifestyle choices influence susceptibility, with diets low in fruits and vegetables and high in saturated fats linked to higher risk. Chronic inflammation, often tied to prostatitis, may further increase susceptibility to prostate cancer. This review article explores the complex realm of prostate cancer, providing insights into its occurrence, factors that elevate risks, and the fundamental factors that play a role in its emergence and how we can prevent it.

## Introduction and background

Situated beneath the bladder, the prostate gland serves as an adjunct reproductive organ in males. Its principal function involves augmenting seminal fluid with vital secretions and preserving the viability of spermatozoa. The fully developed human prostate can be categorised into central, transitional, and peripheral zones. The majority of prostate cancer cases, over 95%, are adenocarcinomas, primarily originating from acinar cells, with a small proportion having a ductal origin. Nearly 80% of prostate adenocarcinomas arise from the luminal cells, with a lower occurrence in basal epithelial cells within the peripheral regions that make up more than 70% of the entire prostate tissue [1]. In 2020, prostate cancer ranked as the second most prevalent cancer, with approximately 1.4 million new cases worldwide. It also stood as the fifth leading cause of cancer-related mortality among men, resulting in approximately 375,000 deaths, and in more than half of the world's nations (112 out of 185), it holds the distinction of being the most commonly diagnosed cancer among men [2].

In its early stages, prostate cancer frequently remains symptomless. Typically, the majority of prostate cancers originate in the peripheral region of the prostate gland. Consequently, for symptoms to become apparent, the cancer must reach a size where it exerts pressure on the urethra or in some cases bone pain from the metastasis might be the initial presenting symptom of advanced prostate cancer. However, any individual aged 50 or above who presents with lower urinary tract symptoms, erectile dysfunction, or hematuria should consider the possibility of prostate cancer [3]. On the other hand, when dealing with advanced metastatic prostate cancer, patients may encounter additional symptoms unrelated to urinary problems. These may include fatigue, bone pain, and unexplained weight loss [4].

The occurrence of prostate cancer is closely linked to age, as per data from the UK's Office for National Statistics. Incidence rates are notably low in individuals below the age of 50 and progressively surge as men reach the age of 50 and beyond, peaking in those aged 90 and older [5]. Moreover, African-American males experience a higher incidence of prostate cancer, often with earlier diagnosis, more aggressive forms of the disease, and increased mortality rates compared to Caucasian males [6].

Prostate cancer is a complex condition influenced by various elements such as age, genetics, lifestyle, and hormonal factors. The epidemiology of this disease highlights significant variations in its occurrence, underscoring the importance of tailored prevention and screening initiatives. A deeper grasp of prostate cancer's origins is also vital, not only for enhancing prevention but also for early detection and treatment approaches. Ongoing research into the intricate interplay of these factors offers hope for reducing the impact of this disease and enhancing the quality of life for those it affects. This literature review, while not following a systematic approach, concentrates on pinpointing the fundamental origins and elements that elevate the risk of prostate cancer and how we can prevent this cancer from increasing in number.

## Review

Epidemiology and incidence**

**Figure 1:**
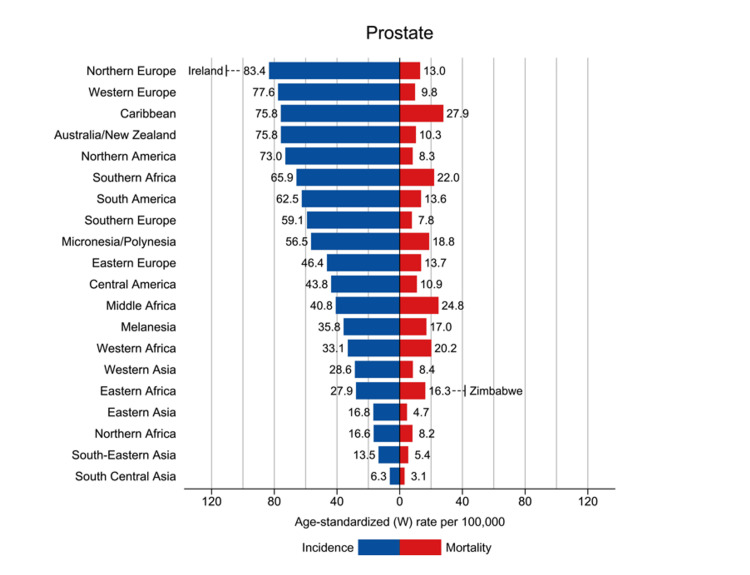
The incidence and mortality rates of Prostate cancer by region during 2020 Source: Reference [2], GLOBOCAN 2020. Correspondence was established with the primary source, culminating in the acquisition of authorization to incorporate the aforementioned figure within the confines of the review.

According to the Global Cancer Incidence, Mortality, and Prevalence (GLOBOCAN) data, prostate cancer (PCa) assumed the second-most prevalent cancer status and ranked as the fifth principal contributor to cancer-related mortality among men in the year 2020, constituting an estimated caseload of nearly 1.4 million fresh diagnoses and approximately 375,000 fatalities distributed across the global landscape. Strikingly, a conspicuous disparity emerged between developed and transitioning nations, as the incidence of prostate cancer registered a notable threefold difference, with rates standing at 37.5 per 100,000 in the former and 11.3 per 100,000 in the latter category. Paradoxically, the mortality rates demonstrated a comparatively narrower range of variation, revealing figures of 8.1 per 100,000 in developed regions and 5.9 per 100,000 in those transitioning (Figure 1) [2].

**Figure 2:**
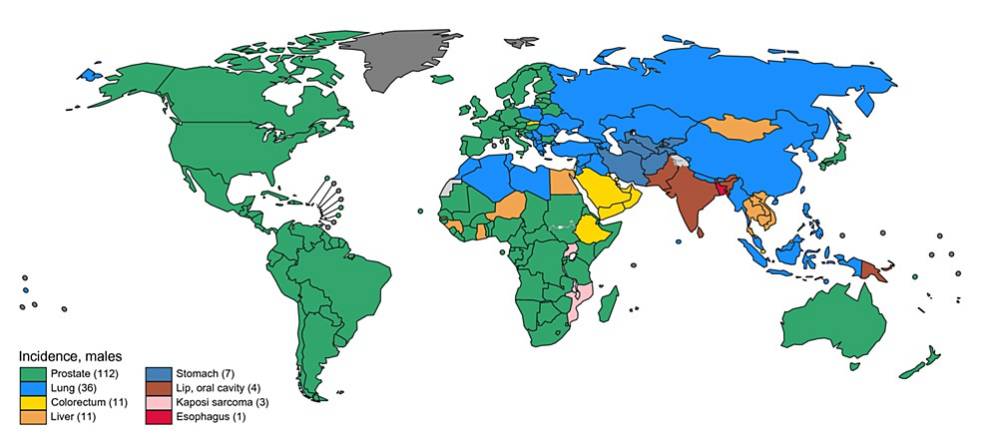
The primary cancer incidence among men in each country in 2020 as per global cancer incidence, mortality, and prevalence (GLOBOCAN) data Source: Reference [2], GLOBOCAN 2020. Correspondence was established with the primary source, culminating in the acquisition of authorization to incorporate the aforementioned figure within the confines of the review.

The condition firmly solidified its status as the most commonly detected malignancy in the male population of a significant majority, specifically 112 out of 185 countries globally (Figure 2). Egregious heterogeneity characterises the incidence rates of prostate cancer, spanning a considerable spectrum from 6.3 to 83.4 per 100,000 men across diverse geographic regions. These contrasting figures showcased the highest incidence in Northern and Western Europe, the Caribbean, Australia/New Zealand, Northern America, and Southern Africa, while conversely, the lowest incidence rates were encountered in the continents of Asia and Northern Africa [2].

For example, while roughly 75% of men in the United States are initially diagnosed with localised-stage prostate cancer, there has been a notable uptick in both the quantity and proportion of men receiving diagnoses of prostate cancer in its distant stages. Although survival rates for distant-stage prostate cancer have shown improvement, it remains that less than one-third of men survive for five years following their diagnosis [7]. In the year 2020, the global landscape of prostate cancer saw an estimated influx of over 1,414,000 new cases, with an age-standardised rate (ASR) incidence of 31 per 100,000 individuals, corresponding to a lifetime cumulative risk of 3.9%. Notably, Northern Europe exhibited the most elevated all-age incidence of ASR at 83, while conversely, the lowest ASR was observed in the region of South-Central Asia, standing at a mere 6.3. During the same period, the worldwide toll from prostate cancer manifested in over 375,000 estimated deaths, with an overarching mortality ASR of 7.7 per 100,000. This rate was found to peak in the Caribbean at 28, whereas it reached its nadir in South-Central Asia at a remarkably low 3.1 [2,8].

On the other hand, Asia has typically been regarded as a region with low incidence rates of prostate cancer, which might be related to the utilization of screening protocols and the meticulous surveillance of data that are intricately connected and could wield a significant influence on the observed incidence rate; however, there has been a swift and notable upswing in both the incidence and mortality of this disease across the continent. Significantly divergent epidemiological characteristics have been identified in various Asian sub-regions, with incidence rates and the ratio of mortality to incidence being linked to the Human Development Index. While Japan and Israel witnessed a decline in prostate cancer mortality between 2007 and 2016, Thailand, Kyrgyzstan, and Uzbekistan experienced an upward trajectory in mortality during the same timeframe [9]. The occurrence of prostate cancer was more prevalent among men residing in urban areas, and the mortality trends appeared to exhibit certain variations based on the specific definitions applied to categorize rural and urban populations, as well as differences in demographic variables and the timeframes considered. Nonetheless, there is compelling evidence indicating that following the introduction of prostate-specific antigen testing, mortality rates tended to rise among men residing in rural areas who had been diagnosed with prostate cancer [10].

Prostate cancer stands as a prevalent malignancy predominantly afflicting the elderly male demographic, a malady whose frequency escalates with advancing age and manifests noteworthy geographical heterogeneity. Even in scenarios where age-specific incidence rates maintain a semblance of constancy, the spectre of prostate cancer looms large, inexorably expanding in absolute terms by virtue of a progressively ageing populace. The consequence of this demographic shift portends substantial economic ramifications for the future, particularly within Westernised societies, where any temporal oscillations in risk merely serve to compound the burgeoning predicament. However, while the incidence of prostate cancer continues its ascent, the risk of succumbing to this ailment has shown a proclivity for immutability in several countries throughout the span of the present century. It behoves us to consider that the augmented incidence may, in part, be attributed to the ascendancy of transurethral resection of prostate procedures or the refinement of screening methodologies, which have ushered in an era of heightened prostate cancer detection and reporting [11]. Likewise, in Spain, while the general incidence rates of prostate cancer appeared to level off between 2002 and 2010 in Navarra, there were distinct variations in trends when considering different age brackets. Specifically, there was an upward trajectory in cases among men aged 45 to 74 years, juxtaposed with a decline in the 75 and older age group. It's noteworthy that a reduction in mortality rates has been evident in both of these age categories since approximately 1995. It's essential to recognise that forthcoming alterations in the utilisation of the prostate-specific antigen test for screening may wield an influence on the trajectory of prostate cancer patterns in the future [12].

Etiology and risk factors 

*(1) Age*****

Age is a firmly established factor that heightens the risk of developing PCa. Recent cancer statistics in the United States demonstrate that the likelihood of PCa escalates from 1.8% in men aged 60 to 69 years to 9.0% in men aged 70 years and beyond, resulting in a lifetime risk of 12.5%. Post-mortem studies have revealed that a substantial 40% of men over 60 years old who haven't undergone screening exhibit prostate cancer, a figure that rises to 60% in those aged over 80 years. It's noteworthy that a significant 32% of these cases fall into the International Society of Urological Pathology grade group ≥2 categories [13,14].

**Figure 3:**
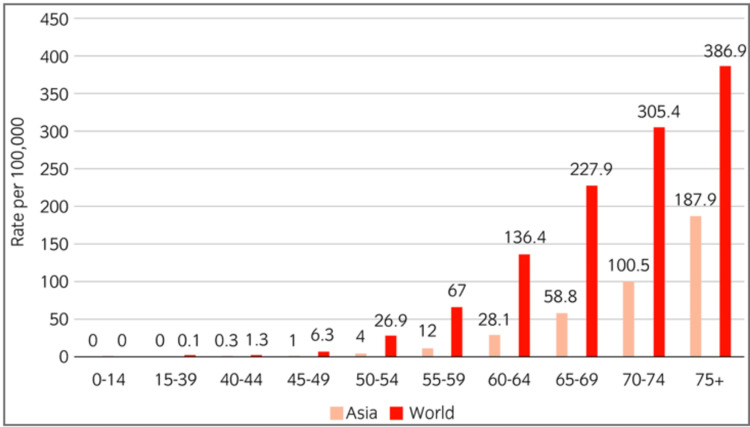
Epidemiology of prostate cancer in Asian countries based on age Source: Reference [15] Correspondence was established with the primary source, culminating in the acquisition of authorization to incorporate the aforementioned figure within the confines of the review.

The prevalence of PCa exhibits a notable proclivity to manifest at a heightened rate among the elderly male demographic. This age-related predisposition is concomitantly and positively correlated with age, exerting a discernible influence on the incidence of both clinically apparent PCa and the prevalence of latent, asymptomatic PCa cases. Figure 3, a graphical representation, meticulously delineates the nuanced intricacies of PCa incidence, stratified by various age brackets, within both the Asian and global populations. Intriguingly, although the incidence of PCa in Asian men tends to maintain a relatively lower profile compared to the global average, a conspicuous and incremental upswing in PCa occurrence becomes conspicuously evident as one progresses through the age groups in both the Asian and global cohorts. The underlying etiology behind this phenomenon, where PCa incidence experiences an escalation within the Asian milieu, is a multifaceted conundrum. It could plausibly be attributed to the remarkable expansion of life expectancies observed in several Asian regions over the past few decades, wherein a confluence of lifestyle factors, healthcare advances, and socio-economic variables intersect to shape the epidemiological landscape of this malignancy [15].

Age exhibits a robust correlation with the risk of developing prostate cancer. Prostate cancer is an infrequent occurrence in men under the age of 40. The incidence of prostate cancer experiences a substantial surge after the age of 55, mirroring a pattern seen in various other epithelial cancers. This age-related trend is observable not only on a global scale but also within regions characterised by varying levels of development. It's worth noting that in 2012, 10% of prostate cancer diagnoses in the United States pertained to men below the age of 55, and this early-onset form of prostate cancer may have a unique origin and clinical presentation. The practice of prostate-specific antigen (PSA) screening confers an advantage of approximately a decade in terms of early detection, as it allows for the identification of prostate cancer prior to the onset of symptoms. In the wake of the adoption of PSA screening in the United States, the average age at which prostate cancer is diagnosed has shifted to an earlier point, currently standing at 66 years of age [16].

On the other hand, the designation of what constitutes "young-age prostate cancer" appears somewhat arbitrary. The upper age limit defining this category, as found in the literature, varies between 50 and 55 years. Epidemiological data from both the UK and the USA indicate that mortality resulting from prostate cancer in individuals under 55 years of age is exceedingly rare, but it experiences a notable upsurge in men aged over 55. Conversely, post-mortem examinations reveal a considerable prevalence of dormant prostate cancer in individuals in their forties and fifties, with the potential for detection through random prostate biopsies triggered by elevated prostate-specific antigen (PSA) levels. The European and North American urology associations have recommended PSA testing, particularly for men over the age of 55. As a consequence of this recommendation, it is probable that in the era of PSA testing, a larger proportion of prostate cancer cases diagnosed in European and American men under the age of 55 exhibit symptomatic features compared to those in men over 55 [17].

*(2) Ethnicity and Race*****

PCa displays a significant variation in its occurrence, impact on health, and fatality rates across various racial and ethnic populations. Among these groups, African-American men have some of the highest PCa rates globally. The causes behind these disparities are multifaceted, involving differences in access to screening and treatment, varying exposure to PCa risk factors, diversity in genetic susceptibility, and the complexity of other biological determinants. Currently, the contribution of environmental factors to PCa disparities appears to be relatively limited, and our comprehension of how these factors exert distinct effects in different racial or ethnic groups remains restricted. In the absence of more comprehensive data, it is increasingly evident that environmental determinants do not play a substantial role in the significant PCa disparities observed [18].

In contrast, prostate cancer boasts one of the highest heritabilities among major cancers, with a multitude of PCa susceptibility genes identified. Certain genetic loci associated with PCa risk, notably those located at chromosome 8q24, demonstrate consistent effects across diverse racial and ethnic cohorts studied thus far. However, the replication of these susceptibility loci remains constrained across varying racial and ethnic backgrounds, warranting further investigation. It is increasingly evident that disparities in access to healthcare services exert a profound influence on PCa disparities [18].

**Figure 4:**
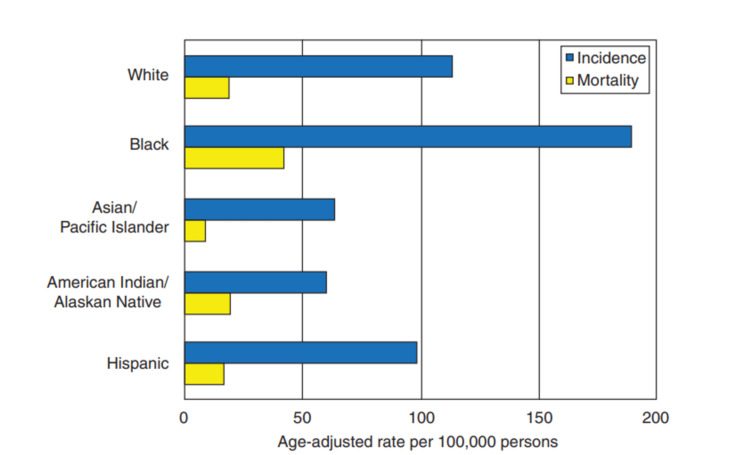
Incidence and mortality rates for prostate cancer per 100,000 individuals categorized by race/ethnicity in the United States These rates are during the period from 2010 to 2014 and have been adjusted to account for age differences among the groups. Source: Reference [16] Correspondence was established with the primary source, culminating in the acquisition of authorization to incorporate the figure mentioned above within the confines of the review.

Significant disparities exist in both the occurrence and fatality rates of prostate cancer among diverse racial and ethnic demographics (as depicted in Figure 4). For instance, there is a notable threefold variance in the incidence rates of prostate cancer when considering various racial and ethnic groups in the United States, with the highest prevalence observed among black men. Furthermore, the number of deaths attributed to prostate cancer is 2.4 times higher among black men compared to white men within the United States. Conversely, prostate cancer incidence and mortality rates exhibit lower figures among Asian/Pacific Islanders, American Indian/Alaskan Natives, and Hispanic men when contrasted with non-Hispanic white men [16].

(3) Hormones 

The prostate gland is under the influence of androgens, which has sparked enduring interest in understanding the role of androgens in the development of prostate cancer. While there is substantial evidence supporting a hormonal basis for prostate cancer, it is primarily indirect and based on inference. A significant challenge in establishing a cause-and-effect relationship lies in the ongoing difficulties associated with accurately measuring the specific exposure of human tissues to naturally produced steroid hormones. The substantial differences in prostate cancer incidence across international and racial-ethnic groups, compounded by the influence of migration on patterns of risk, have prompted the notion that genetic factors are pivotal in determining an individual's susceptibility to prostate cancer. We are in the process of constructing a polygenic model for the development of prostate cancer, with a focus on a series of genes involved in various aspects of androgen biosynthesis, transportation, and metabolism [19].

The etiological framework of PCa extends to encompass an array of estrogenic influences, which encompass a spectrum of sex hormones. These hormones span synthetic and endogenous origins, and they even include compounds derived from fungal and botanical sources. This family of sex hormones exhibits the intriguing capability to traverse the hydrophobic barriers of cell membranes, facilitating their interaction with a constellation of membrane-associated receptors as well as their engagement with intracellular estrogen receptors residing in the nucleus. This intricate interplay subsequently initiates a cascade of gene expression alterations across various organ systems. Remarkably, the role of estrogens as contributors to PCa development and progression remains somewhat underappreciated. This underscores a significant knowledge gap that invites further exploration. Delving deeper into this subject matter not only holds the potential to unveil hitherto unrecognized environmental influencers but also promises to unveil novel therapeutic targets for the efficacious management of prostate cancer. The multifaceted interplay between these estrogenic compounds and PCa pathogenesis represents a compelling avenue for advanced research, one that may have profound implications for our understanding of this complex malignancy and the development of innovative treatment modalities [20].

A plethora of scientific investigations has meticulously cataloged a multitude of contributory factors in the intricate pathogenesis of prostate cancer. These factors encompass the intricate landscape of hormonal imbalances, marked by discernible alterations in androgens and estrogens, the complex interplay of obesity, the weighty influence of a familial history of the disease, and the regulatory sway of growth factors. Within this multifaceted milieu, insulin, a hormone classically associated with metabolic regulation, has emerged as a noteworthy contender. Its involvement in the genesis of several malignancies, including breast and bladder cancers, has been unequivocally recognized. The mechanistic underpinnings of insulin's role in elevating cancer risk are multifaceted and encompass its far-reaching impact on fundamental cellular processes. Specifically, insulin exerts influence over critical phenomena such as cellular proliferation, differentiation, and apoptosis. Recent scientific endeavors, spanning the past decade, have marshaled a wealth of convergent evidence from both epidemiological and clinical research. This growing body of knowledge robustly affirms the pivotal role of insulin in the initiation and subsequent neoplastic progression of prostate cancer. Within the burgeoning field of prostate cancer research, various intricate mechanisms have been proffered to explicate the plausible causal link between insulin and this malignancy. These encompass a spectrum of factors, including the sympathoexcitatory effects of insulin, its modulation of sex hormone metabolism, the intricate entanglement of insulin in the insulin-like growth factor pathway, its involvement in signaling transduction cascades, and its consequential connections to dyslipidemia. These facets collectively paint a rich and complex tapestry of interactions, offering a deeper understanding of the intricate relationships between insulin and the pathogenesis of prostate cancer [21].

*(4) Genetics*****

Recent discoveries underscore the presence of distinct prostate cancer biology among men originating from diverse ethnic and racial backgrounds. This distinctiveness is exemplified by marked disparities in the occurrence of erythroblast transformation-specific-related gene (ERG) oncogenic activation, a critical driver gene that has been extensively studied in prostate cancer research. A comprehensive worldwide analysis of ERG alterations consistently demonstrates that individuals of European descent exhibit the highest frequencies of these genetic modifications, while those of African or Asian heritage manifest significantly lower rates of ERG alterations. It's imperative to approach this data interpretation cautiously, considering the potential influence of variations in assay techniques, types of specimens, and disparities in ethnic and geographic classifications. The endeavor to comprehensively examine cancer-related molecular changes on a global scale holds both promising prospects and formidable challenges, necessitating effective solutions to fully harness the potential of precision medicine for all individuals affected by cancer [22].

The connection between genetic variations in androgen pathway genes and the risk of prostate cancer is intricate, marked by inconsistent findings. The origins of this inconsistency within specific genotype-phenotype associations are challenging to pinpoint and may stem from a combination of biological, statistical, and technical factors. Nevertheless, there are promising developments in the field that extend beyond isolated gene investigations. Initiatives like large-scale single nucleotide polymorphism studies across entire genomes and collaborative efforts spanning multiple nations offer substantial potential for future research, illuminating the more intricate interplays at play in this complex relationship [23].

Single nucleotide polymorphisms (SNPs), which denote variations in DNA sequences marked by a single nucleotide distinction within related strands, represent the most prevalent genetic variations within the human genome. SNPs manifest randomly throughout the genome and are notably prevalent in regions of DNA that do not encode proteins. In the mid-2000s, a groundbreaking research approach known as the genome-wide association study was introduced, enabling the sequential discovery of genetic variants [15]. This method has been instrumental in identifying over 100 published SNPs associated with prostate cancer. In a large-scale, multiethnic genome-wide association study that encompassed 7783 cases and 38,595 controls (comprising 80.3% non-Hispanic white, 4.9% African American, 7.0% East Asian, and 7.8% Latino individuals), it was revealed that risk scores aggregated across all 105 known SNPs exhibited significance across all ethnic groups [24]. Nevertheless, the magnitude of this association displayed substantial variations among ethnicities, possibly due to differing frequencies of risk alleles, notably prominent in East Asian and African American populations [15].

(5) Family History 

Substantial evidence derived from familial investigations underscores the influential role of a family history of prostate cancer in shaping an individual's risk of developing the disease. In comparison to individuals lacking a positive family history, those with a father or brother who has been diagnosed with prostate cancer face a heightened risk, approximately two to three times greater, of receiving their own diagnosis. This risk escalates nearly ninefold for those who have both a father and a brother affected. A parallel association exists concerning the risk of developing lethal prostate cancer. Individuals with a father or brother who succumbed to prostate cancer encounter a twofold increased risk of death from the same condition in contrast to those diagnosed with prostate cancer but devoid of a family history. Moreover, insights from twin studies emphasize that a significant proportion of the familial clustering of prostate cancer can be attributed to shared genetic factors, with a heritability estimate as high as 57% [16].

For sure a prominent risk factor for PCa is the familial history of the ailment. So far, germ-line mutations within the BRCA2 gene, which is typically linked to breast cancer predisposition, have emerged as the most significant genetic events associated with an elevated PC risk, particularly notable with an 8.6-fold increase in men aged 65 or younger. Although the precise mechanisms underlying the influence of BRCA2 on prostate tumorigenesis remain unclear, detrimental mutations in the BRCA2 genes have been linked to more aggressive forms of the disease and unfavorable clinical outcomes [4,25,26].

When examining the interplay between family history and race in the context of prostate cancer, it's noteworthy that a family history of prostate cancer did not exhibit a substantial correlation with the occurrence of prostate cancer, irrespective of its grade, in both black men and non-black men. This was confirmed through both age-adjusted and multivariable analyses. It's important to point out that among black men, a family history of prostate cancer showed a relatively stronger link with an increased likelihood of being diagnosed with high-grade prostate cancer compared to non-black men. However, it's crucial to highlight that this racial distinction did not reach statistical significance, and there was no observable interaction between family history of prostate cancer and race in predicting the probability of high-grade prostate cancer. Furthermore, the lack of information about a family history of prostate cancer did not yield any association with prostate cancer, whether it was of low or high grade, in the entire cohort, or when examined within different racial groups [27].

*(6) Diet* 

Increased attention has been directed toward understanding the impact of diet and nutrients on the development and advancement of PCa. Animal studies have underscored the involvement of specific nutrients, encompassing elements like fats, proteins, carbohydrates, and vitamins (such as vitamins A, D, and E), as well as polyphenols, in the processes of PCa initiation and progression. These mechanisms include their influence on inflammation, antioxidant properties, and interactions with sex hormones [28].

Nevertheless, clinical studies have presented divergent findings, making it challenging to definitively identify which nutrients exert a beneficial or detrimental effect on PCa incidence and progression. The relatively modest effects of individual nutrients, combined with intricate interactions among various nutrients, have yielded conflicting outcomes within clinical studies conducted among individuals with diverse dietary backgrounds. Hence, it becomes imperative to evaluate the impact of dietary patterns that holistically encapsulate nutrient intake. It is widely believed that adopting a wholesome dietary pattern, characterized by a reduced consumption of meat and an elevated intake of vegetables, may play a role in the prevention of PCa and other lifestyle-related diseases. However, it is noteworthy that there is currently insufficient empirical evidence to unequivocally substantiate this belief [28].

Various dietary and lifestyle elements seem to influence the advancement of prostate cancer. Some well-recognized, broadly recommended lifestyle practices, including abstaining from smoking, sustaining a healthy body weight, and engaging in regular, vigorous physical activity, also seem to exert an impact on the progression of prostate cancer. Furthermore, specific dietary components like tomato sauce or lycopene, cruciferous vegetables, sources of healthy vegetable fats, and even coffee, may potentially contribute to diminishing the risk of prostate cancer progression [29].

(7) Body Mass Index

The correlation between obesity and prostate cancer has sparked extensive debate, and despite numerous publications on the subject, the precise nature of this relationship remains uncertain. While some studies have demonstrated a direct connection between obesity and prostate cancer incidence, an equal number of studies have failed to establish any association. Compounding this complexity are recent findings suggesting that obesity in younger men may paradoxically serve as a protective factor against prostate cancer [30]. Several confounding factors contribute to the ambiguity, including the absence of a consistent correlation between body mass index (BMI), a common measure of central obesity, and the timing of BMI measurements concerning diagnosis, such as whether they were taken before or after the diagnosis, as well as during young or advanced adulthood. The evidence regarding increased BMI as a risk factor for prostate cancer is characterized by a lack of clarity. However, it is becoming increasingly evident that obesity is linked to more unfavorable disease prognoses, diminished post-surgical outcomes, and heightened mortality rates related to prostate cancer, irrespective of margin status. From a biological standpoint, several potential mechanisms have been proposed to explain how obesity might contribute to prostate cancer development and progression. These mechanisms include low testosterone levels, elevated estrogen levels, the presence of diabetes or metabolic syndrome, heightened circulating levels of insulin-like growth factor-one (IGF-1), increased leptin levels, reduced adiponectin levels, and an augmented consumption of dietary saturated fats [30].

Nevertheless, the mounting body of evidence underscores a compelling association between obesity and various critical dimensions of PCa. This multifaceted relationship encompasses a higher prevalence of aggressive PCa, an increased susceptibility to biochemical failure subsequent to radical prostatectomy or external-beam radiotherapy, an elevated incidence of complications following androgen-deprivation therapy, and an augmented risk of PCa-specific mortality. Curiously, these associations persist alongside the possibility of a comparatively lower overall incidence of PCa among obese individuals. It is important to acknowledge that these findings may be partially influenced by the inherent challenges in the identification and management of PCa in the context of obesity [30,31].

However, delving into the realm of molecular intricacies offers multiple potential mechanisms that could elucidate these connections. Notably, while animal models have demonstrated that weight loss can impede the progression of PCa, the translation of these insights into clinical human trials is an avenue that awaits comprehensive exploration [31].

*(8) Inflammation (Prostatitis)*****

Persistent inflammation has been linked to the development of various solid cancers and may play a contributory role in the initiation of prostate cancer. This supposition is, in part, rooted in the recurrent observation of inflammatory cells within the prostate microenvironment of adult males. Inflammation is closely associated with putative precursor lesions for prostate cancer, notably referred to as proliferative inflammatory atrophy. Inflammation is theorized to drive the process of prostate carcinogenesis through the induction of oxidative stress and the generation of reactive oxygen species, which, in turn, may give rise to genetic mutations. Additionally, the stress imposed by chronic inflammation could trigger epigenetic alterations that facilitate the neoplastic transformation of prostate cells. Proliferative inflammatory atrophy exhibits an enrichment of proliferative luminal epithelial cells of an intermediate phenotype, potentially rendering them susceptible to genomic changes that ultimately lead to the development of prostatic intraepithelial neoplasia and, subsequently, prostate cancer. Insights gleaned from animal studies suggest that inflammatory modifications within the prostate microenvironment can contribute to the reprogramming of prostate epithelial cells, representing a plausible initial step in the process of tumor formation [32].

In the context of the prostate, infections within this organ, often accompanied by disruptions in the epithelial barrier, are posited as pivotal contributors to the establishment of an inflammatory microenvironment. The revelation of a urinary microbiome introduces the possibility of recurrent exposure of the prostate to a diverse array of microorganisms. Hence, current empirical evidence posits that inflammation and atrophy play intricate roles in the genesis of prostate cancer, with the microbiome potentially serving as a source for the consistent exposure of the prostate to an inflammatory milieu that could promote the development and progression of prostate cancer [32,33].

In other words, prostate cancer and chronic prostatitis represent common maladies afflicting the male population. The etiology of both prostate cancer and chronic prostatitis is marked by a multifaceted and heterogeneous array of factors. Notably, both conditions manifest with a distinctive rise in serum prostate-specific antigen levels, which currently serves as the primary diagnostic test for prostate cancer. An underlying commonality is the presence of prostate inflammation, irrespective of its root cause, which emerges as the histopathological hallmark of chronic prostatitis. Broadly, inflammation is recognized as a potential precursor for a range of cancers, and in the specific case of prostate health, the presence of prostatic inflammation is posited as a conceivable contributor to the genesis and progression of prostate cancer [33].

*(9) Pesticides Exposure and Smoking*****

Numerous cancers have been associated with distinct environmental exposures. Extensive research has delved into potential environmental and occupational elements that might be correlated with the risk of PCa. These factors encompass exposure to Agent Orange, engagement in farming, and the use of pesticides, as well as exposure to sunlight and ultraviolet radiation. Additionally, trace minerals employed in the manufacturing processes of items like tires and batteries have been scrutinized for their potential role in prostate cancer risk [34]. As an illustration, individuals who had used the organodithioate insecticide dimethoate at any point exhibited a heightened risk of developing aggressive prostate cancer compared to those who had never used it [35]. On the other hand, although tobacco use has been recognized as one of the leading causes of cancer morbidity and mortality, the role of smoking in the occurrence of prostate cancer has not been established.

*(10) Height*****

A consistent and moderate positive association has been observed between adult height and the incidence of prostate cancer [36]. For instance, greater height has been correlated with an elevated risk of developing localized intermediate-risk prostate cancer. Conversely, being overweight or obese has been linked to decreased risks of localized low- and intermediate-risk prostate cancers [37].

*(11) Vasectomy*****

Vasectomy, a commonly chosen method of male contraception, has garnered attention due to the prevalence of prostate cancer in men. The endeavor to establish a potential cause-and-effect relationship between vasectomy and prostate cancer has been ongoing. While some prior studies have suggested a connection between the two, the body of supporting evidence falls short of establishing a conclusive causal link. Examining the methodologies employed in these studies reveals the presence of potential biases and confounding factors, which could account for some of the observed positive associations. In more recent research endeavors, marked by their inclusion of a wider array of controlling variables and larger sample sizes, the consensus aligns with the absence of a significant association between vasectomy and prostate cancer [38]. 

(12) Syndromes

Metabolic syndrome (MetS) has been associated with various forms of cancer in the human population. Among these, prostate cancer stands as the most prevalent malignancy affecting adult males, often necessitating radical prostatectomy as a treatment approach. Given the intricate interplay of hormonal and metabolic disturbances characterizing MetS, there has been a hypothesis positing its potential involvement in the onset and progression of PCa. Numerous studies have been conducted to explore the potential links between MetS and the risk of developing prostate cancer, as well as the impact of this syndrome on the clinical outcomes of individuals already diagnosed with PCa who undergo radical treatment [39]. Also, Lynch syndrome is considered a relatively uncommon genetic disorder, primarily attributed to mutations found within several genes, including notable ones such as MLH1 and MLH2. It's essential to recognize that individuals with Lynch syndrome confront an augmented risk of developing an array of cancers, which encompasses prostate cancer, further underlining the significance of proactive monitoring and potential preventive measures for these individuals [4].

Prevention and protective factors

When considering the hazards and origins of PCa, the approach to preventing this ailment would involve addressing these root causes. For instance, numerous preliminary research and observational inquiries have indicated that dietary adjustments, physical activity, and lifestyle modifications might have a part in alleviating the advancement of the disease, reducing mortality, and lessening the overall disease-related burden for severe and lethal prostate cancer. Augmented consumption of vegetables and fruits, diminished intake of red meat and saturated fats, as well as heightened levels of physical activity, are factors that could potentially correlate with a lowered likelihood of developing the disease and an increase in progression-free, prostate cancer-specific, and overall survival rates [28,29,40]. Likewise, prostate cancer is a disease characterized by multifaceted etiology, which underscores the complexity of its development and progression. Any strategy aimed at addressing the racial and ethnic disparities prevalent in PCa must necessarily consider the intricate interplay of these multifarious factors that collectively impact the risk, outcomes, and disparities associated with this condition [18].

Numerous professional organizations are now advocating against population-based PSA screening. This stance does not oppose the early detection of prostate cancer but rather stems from a reaction to decades of discovering and treating clinically inconsequential prostate cancers, along with the limited impact on mortality [41]. To date, PSA screening has fallen short of meeting the fundamental public health requirement of causing more benefits than harm. The widespread practice of testing and treating has resulted in substantial personal and financial costs, leading to a recent backlash against population screening. The advent of derived or novel diagnostic tests may pave the way for a more rational approach to prostate cancer screening and the selection of men who require closer monitoring or diagnostic biopsies. Meanwhile, the decision to pursue PSA screening should be the outcome of a deliberate and informed discussion between individual men and their healthcare providers. PSA remains a valuable, albeit imperfect, tool for monitoring men following treatment for localized disease or those with advanced prostate cancer [41].

Conversely, the precise causes are largely a mystery, and there are no evident risk factors to pinpoint. Consequently, primary prevention poses a significant challenge. Additionally, conducting large-scale secondary prevention measures, like screening, is complicated. Hereditary prostate cancer has links to germline mutations, accounting for approximately 10% of screened individuals. Presently, the sole method in place for early detection is the prostate-specific antigen test, yet it is regarded as inadequate to substantially enhance prevention and healthcare [42].

## Conclusions

Prostate cancer, the second most common cancer among men globally, emerges as a significant health concern with advancing age. It is essential to grasp the epidemiological and causative factors behind this disease to develop effective prevention strategies and enhance treatment outcomes. Age is a pivotal factor, as the risk escalates in older men. Developed nations witness higher incidence rates, influenced by lifestyle choices and environmental factors. Racial and geographical disparities exist, notably affecting African American men with elevated occurrence rates and more aggressive disease forms. Hormonal dynamics, genetic mutations like BRCA1 and BRCA2, diet, lifestyle, and chronic inflammation play roles in susceptibility. This review explores these facets and strategies to reduce prostate cancer incidence.

## References

[1] Wasim S, Lee SY, Kim J (2022). Complexities of prostate cancer. Int J Mol Sci.

[2] Sung H, Ferlay J, Siegel RL, Laversanne M, Soerjomataram I, Jemal A, Bray F (2021). Global cancer statistics 2020: GLOBOCAN estimates of incidence and mortality worldwide for 36 cancers in 185 countries. CA Cancer J Clin.

[3] (2023). Suspected cancer: recognition and referral. https://www.nice.org.uk/guidance/ng12.

[4] (2023). Cancer research UK : Prostate Cancer. https://www.cancerresearchuk.org/about-cancer/prostate-cancer.

[5] Merriel SW, Funston G, Hamilton W (2018). Prostate cancer in primary care. Adv Ther.

[6] Lillard JW Jr, Moses KA, Mahal BA, George DJ (2022). Racial disparities in Black men with prostate cancer: a literature review. Cancer.

[7] Siegel DA, O'Neil ME, Richards TB, Dowling NF, Weir HK (2020). Prostate cancer incidence and survival, by stage and race/ethnicity - United States, 2001-2017. MMWR Morb Mortal Wkly Rep.

[8] Gandaglia G, Leni R, Bray F (2021). Epidemiology and prevention of prostate cancer. Eur Urol Oncol.

[9] Zhu Y, Mo M, Wei Y (2021). Epidemiology and genomics of prostate cancer in Asian men. Nat Rev Urol.

[10] Obertova Z, Brown C, Holmes M, Lawrenson R (2012). Prostate cancer incidence and mortality in rural men--a systematic review of the literature. Rural Remote Health.

[11] Boyle P, Maisonneuve P, Napalkov P (1996). Incidence of prostate cancer will double by the year 2030: the argument for. Eur Urol.

[12] Etxeberria J, Guevara M, Moreno-Iribas C (2018). Prostate cancer incidence and mortality in Navarre (Spain). An Sist Sanit Navar.

[13] Siegel RL, Miller KD, Fuchs HE, Jemal A (2022). Cancer statistics, 2022. CA Cancer J Clin.

[14] Zlotta AR, Egawa S, Pushkar D (2013). Prevalence of prostate cancer on autopsy: cross-sectional study on unscreened Caucasian and Asian men. J Natl Cancer Inst.

[15] Kimura T, Egawa S (2018). Epidemiology of prostate cancer in Asian countries. Int J Urol.

[16] Pernar CH, Ebot EM, Wilson KM, Mucci LA (2018). The epidemiology of prostate cancer. Cold Spring Harb Perspect Med.

[17] Hussein S, Satturwar S, Van der Kwast T (2015). Young-age prostate cancer. J Clin Pathol.

[18] Rebbeck TR (2018). Prostate cancer disparities by race and ethnicity: from nucleotide to neighborhood. Cold Spring Harb Perspect Med.

[19] Ross RK, Coetzee GA, Pearce CL (1999). Androgen metabolism and prostate cancer: establishing a model of genetic susceptibility. Eur Urol.

[20] Dobbs RW, Malhotra NR, Greenwald DT, Wang AY, Prins GS, Abern MR (2019). Estrogens and prostate cancer. Prostate Cancer Prostatic Dis.

[21] Nandeesha H (2009). Insulin: a novel agent in the pathogenesis of prostate cancer. Int Urol Nephrol.

[22] Sedarsky J, Degon M, Srivastava S, Dobi A (2018). Ethnicity and ERG frequency in prostate cancer. Nat Rev Urol.

[23] Mononen N, Schleutker J (2009). Polymorphisms in genes involved in androgen pathways as risk factors for prostate cancer. J Urol.

[24] Ahmed M, Eeles R (2016). Germline genetic profiling in prostate cancer: latest developments and potential clinical applications. Future Sci OA.

[25] Song WH, Kim SH, Joung JY, Park WS, Seo HK, Chung J, Lee KH (2017). Prostate cancer in a patient with a family history of BRCA mutation: a case report and literature review. J Korean Med Sci.

[26] (1999). Cancer risks in BRCA2 mutation carriers. J Natl Cancer Inst.

[27] Jenkins KR, Oyekunle T, Howard LE, Wiggins EK, Freedland SJ, Allott EH (2021). Family history of prostate cancer and prostate tumor aggressiveness in black and non-black men;results from an equal access biopsy study. Cancer Causes Control.

[28] Matsushita M, Fujita K, Nonomura N (2020). Influence of diet and nutrition on prostate cancer. Int J Mol Sci.

[29] Peisch SF, Van Blarigan EL, Chan JM, Stampfer MJ, Kenfield SA (2017). Prostate cancer progression and mortality: a review of diet and lifestyle factors. World J Urol.

[30] O'Malley RL, Taneja SS (2006). Obesity and prostate cancer. Can J Urol.

[31] Allott EH, Masko EM, Freedland SJ (2013). Obesity and prostate cancer: weighing the evidence. Eur Urol.

[32] Sfanos KS, Yegnasubramanian S, Nelson WG, De Marzo AM (2018). The inflammatory microenvironment and microbiome in prostate cancer development. Nat Rev Urol.

[33] Sandhu JS (2008). Prostate cancer and chronic prostatitis. Curr Urol Rep.

[34] Mullins JK, Loeb S (2012). Environmental exposures and prostate cancer. Urol Oncol.

[35] Pardo LA, Beane Freeman LE, Lerro CC (2020). Pesticide exposure and risk of aggressive prostate cancer among private pesticide applicators. Environ Health.

[36] Bjerregaard LG, Aarestrup J, Gamborg M, Lange T, Tjønneland A, Baker JL (2016). Childhood height, adult height, and the risk of prostate cancer. Cancer Causes Control.

[37] Jochems SH, Stattin P, Häggström C, Järvholm B, Orho-Melander M, Wood AM, Stocks T (2020). Height, body mass index and prostate cancer risk and mortality by way of detection and cancer risk category. Int J Cancer.

[38] Nutt M, Reed Z, Köhler TS (2016). Vasectomy and prostate cancer risk: a historical synopsis of undulating false causality. Res Rep Urol.

[39] Morlacco A, Zattoni F, Soligo M, Lami V, Iafrate M, Zanovello N, Dal Moro F (2022). Metabolic syndrome and prostate cancer treatment. Panminerva Med.

[40] Ballon-Landa E, Parsons JK (2018). Nutrition, physical activity, and lifestyle factors in prostate cancer prevention. Curr Opin Urol.

[41] Pezaro C, Woo HH, Davis ID (2014). Prostate cancer: measuring PSA. Intern Med J.

[42] Cozzi G, Musi G, Ferro M, Cioffi A, de Cobelli O, Corso G (2022). Progress in prostate cancer prevention. Eur J Cancer Prev.

